# The effects of thermal treatment on the bacterial community and quality characteristics of meatballs during storage

**DOI:** 10.1002/fsn3.2026

**Published:** 2020-12-02

**Authors:** Ran Li, Chong Wang, Guanghong Zhou, Chunbao Li, Keping Ye

**Affiliations:** ^1^ Key Laboratory of Meat Processing and Quality Control Ministry of Education Nanjing China; ^2^ Jiangsu Collaborative Innovation Center of Meat Production and Processing, Quality and Safety Control Nanjing Agricultural University Nanjing China

**Keywords:** bacterial community, meatball, metabolic pathway, physicochemical quality, thermal treatment

## Abstract

Thermal treatment is a widely applied food processing technology in the meat industry due to its convenience. However, the interpretation of the changes in the bacterial community and quality properties in the thermal processed meat products have not been well established. Therefore, the effects of thermal treatment on the quality characteristics and bacterial communities in meatballs during storage at 4°C were investigated, which will provide a more comprehensive understanding of the influence of thermal treatment on the meat quality. Thermal treatment (121°C, 15 min) decreased the initial total viable bacterial counts by 2.1 log CFU/g and the diversity of the initial bacterial communities in meatballs. Compared with the thermal treatment group, a significantly more rapidly increasing trend of total volatile basic nitrogen and a decreasing trend of pH were observed in the control group. At the end of storage, the bacterial community was dominated by *Streptococcus*, *Acinetobacter* and *Pseudomonas* in the thermal treatment meatballs, whereas *Pseudomonas*, *Pantoea*, and *Serratia*. dominated the bacterial community of the control group. Besides, the predicted metabolic pathways revealed high levels of carbohydrate, amino acid, and lipid metabolism in the control group. This finding could contribute to a deep understanding of the influence of thermal treatment on the meat quality. Moreover, these results could provide a theoretical foundation for the development of alternative and novel nonthermal processing technologies for use in the meat industry.

## Graphical abstract

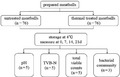

## 1 INTRODUCTION

Meat is a perishable food that is an ideal substrate for the growth of spoilage microorganisms (Huang et al., 2020; Nychas et al., 2008; Qi et al., 2015). Thus, it is essential to apply strict hygiene procedures and adequate processing technologies to delay the process of meat spoilage and maintain meat quality and safety (Xiong, 2017). Heat processing, commonly including pasteurization (60–85°C) and commercial sterilization (generally at above 121°C), is regarded as a pivotal method for the preservation of meat products as it could not only guarantee the edible safety, but also extend the shelf‐life, and among which, commercial sterilization is the most commonly used one (Lyng et al., 2019; Misra & Jo, 2017; Wu et al., 2020). Thermal processing is a unit operation in which foods are heated at a sufficiently high temperature for specific amount of time to destroy vegetative microbial cells, spores and enzymes (Fellows, 2017). However, the texture, taste, flavor, and nutritional value of meat products are significantly deteriorated because of the thermal processing treatment (Barbosa‐Cánovas et al., 2014).

Studies have been performed to analyze the effects of thermal processing on the flavor profile, shelf‐life, texture, and sensory characteristics of meat and meat products (Barbosa‐Cánovas et al., 2014; Wang et al., 2019). For example, Wang et al. (2019) observed a significantly higher peroxide value in a water bath thermal sterilization group than that detected in the control group, which indicated that lipid oxidation in the vacuum‐packaged braised beef was accelerated via water bath thermal sterilization. In addition, Song et al. (2019) showed that thermal processing at 121°C could prolong the shelf‐life but deteriorate the flavor properties of salted duck. In thermal processing of meat products, the change in food structure and loss of texture is common; additionally, lipid oxidation is a major cause of rancidity and off‐flavors during subsequent storage (Misra & Jo, 2017). It is well established that the initial total bacterial counts significantly decreased via thermal processing treatment, whereas the changes of specific bacterial genus and bacterial metabolism in meat products have not been investigated. Currently, some studies have investigated the effect of processing treatments on the bacterial communities in meat and meat products (Han et al., 2020; Nieminen et al., 2012; Wang, Qin, et al., 2020). However, relatively little work has been done on the influence of thermal processing treatment on the bacterial communities in meat and meat products. And the influence of thermal processing on the bacterial metabolism in meat products during storage has not been well detailed.

In the contemporary world, emerging technologies like high hydrostatic pressure, pulsed electric field, ultrasound, ionizing radiation, and cold atmospheric plasma have shown the potential in achieving industrial application in foods (Hernández‐Hernández et al., 2019). However, microbial inactivation of these nonthermal processing technology can be influenced by the initial number of microorganisms, environmental influences, food composition, exposure time, the characteristics of microorganisms present in that food (Horita et al., 2018; Song et al., 2009). Hence, conventional thermal processing processed significant advantages in microbial inactivation than those in nonthermal processing technologies, and thermal treatment is still dominant in food preservation field (Li et al., 2017). Investigations of the effect of thermal treatment on the bacterial communities may provide the theoretical foundation for developing alternative and novel nonthermal processing technologies for use in the meat industry. Therefore, the effects of thermal processing treatment (121°C 15 min) on the quality characteristics and bacterial communities in meatballs during storage at 4°C were investigated, which will provide a more comprehensive understanding of the influence of thermal processing treatment on the meat quality and safety.

## 2 MATERIALS AND METHODS

### 2.1 Sampling and storage

Meatballs (ingredients: pork; horse hoof; shallot; and spices) with an average weight of 180 g/bag were collected from a local meat products processing company. Among the meatballs, seventy‐six bags of meatballs were treated at 121°C for 15 min using a sterilization kettle (SH 800; Jinding Instruments) after they were packaged, which were assigned to the thermal processing group, while the other samples were assigned to the control group. All samples were transferred into chilled insulated boxes and transported to the laboratory within 3 hr. Then, all the samples were stored at 4°C for up to 21 days and analyzed at days 0, 7, 14, and 21.

### 2.2 pH measurements

The pH of the meatball samples was measured according to the method described by Huang et al. (2020) with some appropriate modifications. Briefly, a 3.0 g sample of meatballs was mixed with 30 ml of an ice‐cold solution (pH = 7.0) comprising 5 mM sodium iodoacetate and 150 mM potassium chloride. The mixtures were homogenized using a stomacher (Ultra Turrax T25, IKA) at 4000 g for 30 s. Then, the pH values of meatballs were measured with a pH meter (Hanna HI9025c; Hanna Instruments).

### 2.3 Total volatile basic nitrogen (TVB‐N) assays

The TVB‐N of meatballs was determined according to the method described by Gharibzahedi and Mohammadnabi (2017) and Huang et al. (2020) with some appropriate modifications. Briefly, 10 g of minced meatball sample was homogenized in 100 ml of distilled water at room temperature for 30 min. After filtering, 10 ml of the resulting supernatant was mixed with 10 ml of MgO (10 g/L) and then distilled using a nitrogen apparatus (K1160, HaiNeng Instruments). The TVB‐N of meatball was expressed as mg of N per 100 g of meat.

### 2.4 Total viable counts (TVC)

The total viable counts (TVC) for each sample were obtained using the pour plate technique according to the China National Food Safety Standard methods (Food Microbiology Examination‐Aerobic Plate Count; GB 4789.2–2016). Briefly, 25 g of meatball sample was aseptically transferred to a sterile stomacher bag and homogenized in 225 ml of sterile saline for 2 min in a stomacher (BagMixer 400 VW, Interscience Co.). After performing 1:10 serial dilutions, 1 ml of the suspension from each dilution was inoculated onto plate count agar (PCA, Beijing Luqiao) and incubated at 37°C for 48 hr to allow the total viable counts to be determined. The results were expressed as decimal logarithms of colony‐forming units per gram (LogCFU/g).

### 2.5 Characterization of bacterial communities

#### 2.5.1 DNA extraction and 16S rRNA amplicon sequencing

Total bacterial genomic DNA was extracted from meatball samples according to the method described by Zhang et al. (2017) with some modifications. Briefly, 10 ml of homogenates (obtained in section 2.4) were centrifuged at 13,000 × *g* for 10 min. Subsequently, the pelleted sediment was resuspended in 1 ml of a sterile 0.9% NaCl solution and transferred to centrifuge tubes. DNA was then extracted from the pelleted sediment using a bacterial DNA extraction kit (TIANamp, Beijing Tiangen Co., Ltd.). To break open the cell walls of gram‐positive bacteria, 20 µl of lysozyme (20 mg/ml) was added to each sample. The final DNA concentration and purity were determined using a NanoDrop 2000 UV‐Vis spectrophotometer (Thermo Scientific). DNA quality was checked by 1% agarose gel electrophoresis. The DNA preparations were used for PCR with the primers 341‐F (CCTAYGGGRBGCASCAG) and 806‐R (GGACTACNNGGGTATCTAAT), which target the V3‐V4 region of the 16S rRNA gene. PCR was performed in a final volume of 25 µl containing 12.5 µl of Phusion^®^ High‐Fidelity PCR Master Mix (New England Biolabs), 1 µl of primer, 2 µl of template DNA, and PCR grade water. Thermal cycling consisted of an initial denaturation at 98°C for 1 min followed by 30 cycles of denaturation at 98°C for 10 s, primer annealing at 50°C for 30 s, and extension at 72°C for 60 s, with a final extension step at 72°C for 5 min. The PCR products were mixed in identical ratios and purified using a GeneJET Gel Extraction kit (Thermo Scientific).

#### 2.5.2 Illumina MiSeq sequencing and data processing

Purified amplicons were pooled in equimolar volumes using a TruSeq™ DNA Sample Preparation kit (Illumina Inc.). Pooled library quantitation and paired‐end Illumina MiSeq sequencing (2 × 300 bp) were performed on the Illumina MiSeq platform (Illumina), which was conducted by Lingen Biotechnology Co. Ltd. The raw sequence reads were demultiplexed and quality‐filtered using the Quantitative Insights into Microbial Ecology software (QIIME version 1.9.1) (Caporaso et al., 2010). The sequences were clustered into operational taxonomic units (OTUs) based on UPARSE (version 7.1 http://drive5.com/uparse/) with a 97% identity threshold. These sequencing data were searched against the Gold database (http://drive5.com/uchime/uchime_download.html) using the UCHIME algorithm (http://www.drive5.com/usearch/manual/uchime_algo.html). Principal component analysis (PCA) was performed to analyze the differences in bacterial community composition between samples. Based on the species annotation and abundances of effective OTUs, functional annotations were obtained using the Kyoto Encyclopedia of Genes and Genomes (KEGG) pathway with Tax4Fun v1.0 Asshauer (Asshauer et al., 2015).

### 2.6 Statistical analysis

The data are presented as the means and standard deviations. Differences in mean values for TVB‐N, pH, and TVC were analyzed using one‐way ANOVA implemented in IBM SPSS Statistics 20.

## 3 RESULTS AND DISCUSSION

### 3.1 pH

The pH changes in the thermal processing and control group during refrigerated storage are shown in Figure 2a. The initial pH values of thermal processing and control group were 6.505 ± 0.02 and 6.510 ± 0.01, respectively, which demonstrated that thermal processing treatment has little influence on the initial pH of the meatballs in this study. Wang, Shi, et al. (2020) investigated the effect of different thermal temperatures on the pH of Dezhou‐braised chicken and observed that heat treatment did not significantly alter the initial pH of samples, which was consistent with our results. Although the pH in both the thermal processing and control group decreased gradually throughout the entire storage period, the pH in the control group was significantly lower than that observed in the thermal processing group at the end of storage (*p* < .05). The reason for this result may be that more acid‐forming bacteria were present in the control group, which may have led to a lower pH of the meatballs. The above findings indicated that the use of thermal treatment (121°C, 15 min) could delay the pH decrease in meatballs during storage. Song et al. (2019) studied the pH changes in untreated and thermal processed salted duck during 4°C storage, observing that thermal treatment (121°C, 30 min) could diminish the decreasing rate of pH in the salted ducks, which was in accordance with our findings.

**FIGURE 1:**
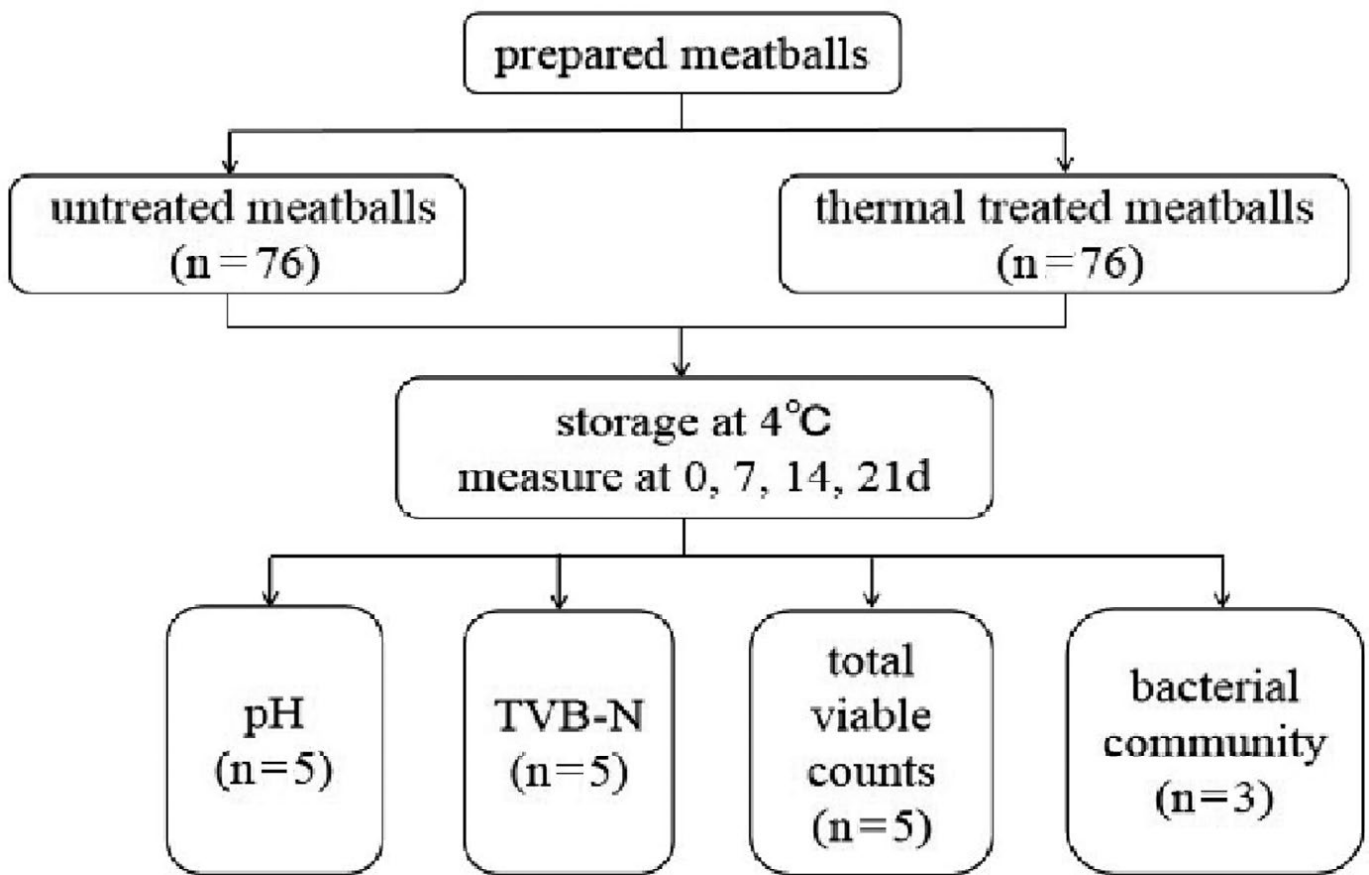
Experimental design and workflow of sample collection

### 3.2 TVB‐N

**FIGURE 2:**
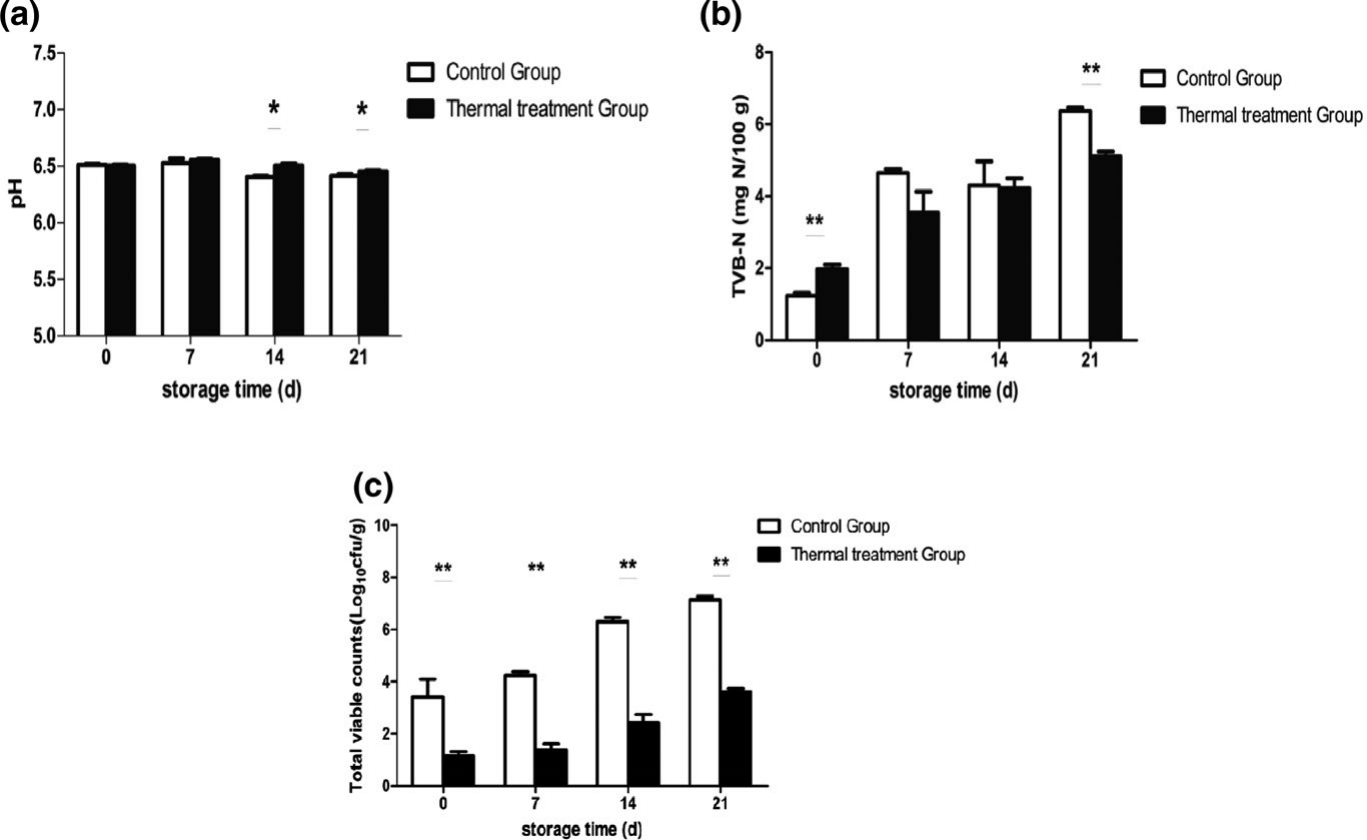
Changes in pH, TVB‐N, and TVC values of thermal treatment (121°C, 15 min) and control groups during storage. (a) pH; (b) TVB‐N; (c) TVC. (*n* = 5). *p < .05, **p < .01

Total volatile basic nitrogen (TVB‐N), which primarily comprises amines and ammonia, is widely used as an indicator of meat spoilage, resulting from the microbial degradation of protein and nonprotein nitrogenous compounds, such as amino acids and nucleotide catabolites (Liu et al., 2013). Figure 2b shows the effect of thermal treatment on the TVB‐N in meatballs during 4°C storage. Compared with the control group, a significantly higher TVB‐N value was observed in the thermal treatment group (*p* < .05), which indicated that the initial TVB‐N in meatballs was significantly influenced by the thermal treatment. These results could be explained by the degradation reactions of proteins being accelerated by the thermal treatment process, which may have produced more amino acids and volatile bases in the thermal treatment group (Guo et al., 2016). The TVB‐N values in both the thermal treatment and control groups displayed a continually increasing tendency during 4°C storage. Moreover, the control group exhibited a more dramatic increasing behavior, which may be due to more active bacterial metabolic activities in the control group. In cooked meat products, the increase in TVB‐N is attributed to bacterial metabolic substances and degradation of protein and other nonprotein nitrogen‐containing compounds, such as ammonia, monoethylamine, dimethylamine, and trimethylamine (Feng et al., 2016; Wang et al., 2017).

### 3.3 TVC

The initial TVC values of thermal treatment and control groups were 1.15 ± 0.15 and 3.41 ± 0.61 log CFU/g (Figure 2c), respectively, which demonstrated that the thermal treatment could kill and reduce approximately 2 log CFU/g in samples. Furthermore, bacteria grew more rapidly in the control group due to a significantly higher initial bacterial concentration. The TVC in the control group reached a maximum level of 7.14 ± 0.11 log CFU/g after 21 days of storage, whereas the TVC of the thermal treatment group was only 3.60 ± 0.13 log CFU/g. Wang et al. (2019) reported that the initial TVC value in vacuum‐packaged braised beef was reduced by 1.6 log CFU/g using the thermal treatment (90°C, 30 min) method. In addition, Song et al., 2019) demonstrated that thermal treatment (121°C, 30 min) could decrease the initial TVC value by 1.6 log CFU/g in salted ducks. The results of this study also illustrated that the use of thermal treatment could effectively reduce the initial TVC of meat products during 4°C storage.

### 3.4 Bacterial richness and diversity

**FIGURE 3:**
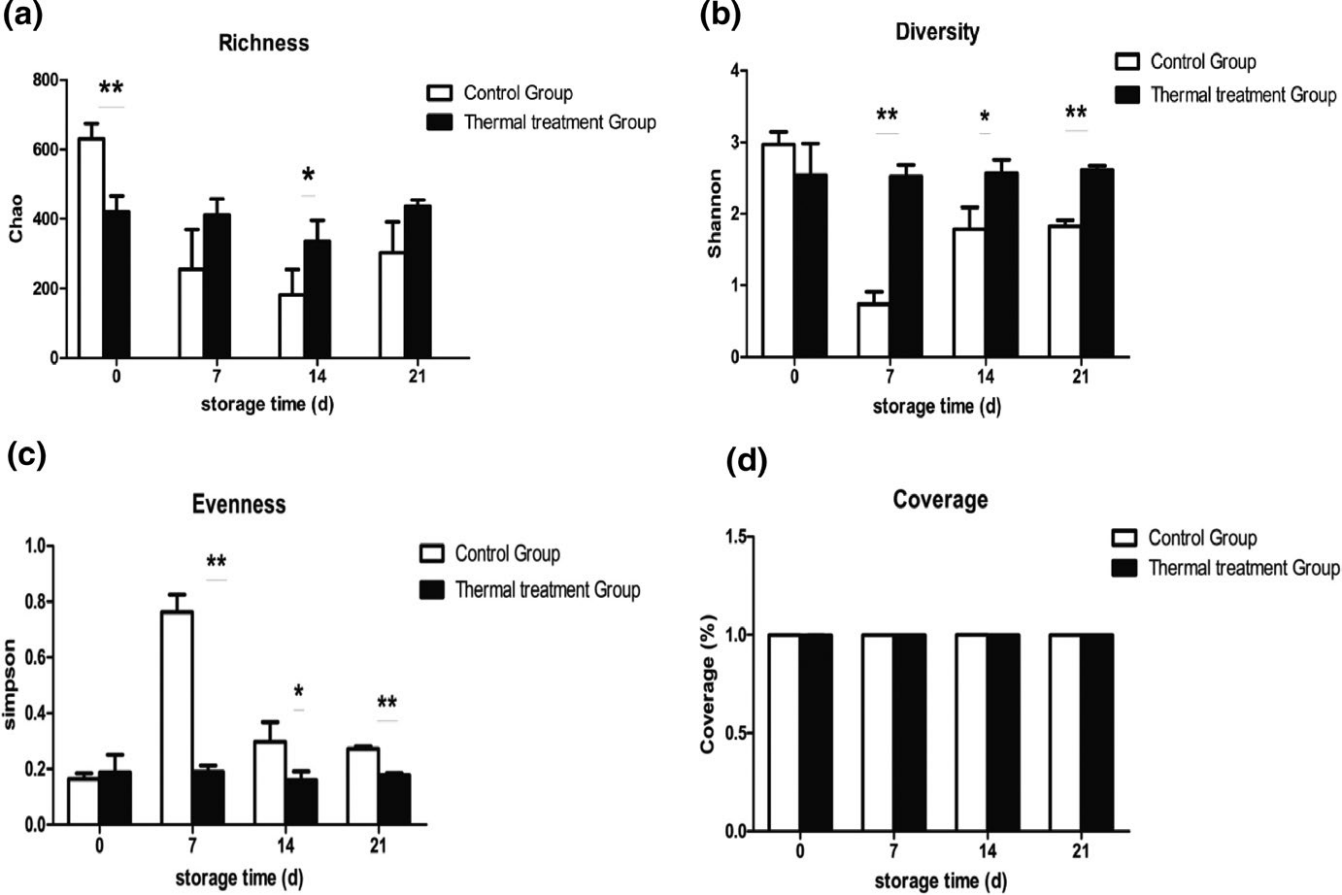
Changes in alpha‐diversity values of thermal treatment (121°C, 15 min) and control groups during storage at 4°C. (a) Chao index value changes; (b) Shannon index value changes; (c) Simpson index value changes; (d) Coverage index value changes. The data are presented as the means ± standard deviation (*n* = 3). *p < .05, **p < .01

Amplicon sequencing of the 16S rRNA gene could provide a comprehensive understanding of the diversity and abundance of bacterial communities in meatballs during refrigerated storage. A total of 1,020,024 high quality effective sequences were obtained by merging and filtering the raw sequences, which was equivalent to an average of 42,501 sequences for each sample. The sequence length in 24 samples was from 407.12–428.77 bp (Table 1). Coverage values for all samples were >99%, which demonstrated that the present sequencing results represented an accurate picture of the microorganisms in the samples. Effective sequences were clustered into 6,969 OTUs using a 97% similarity threshold. The alpha‐diversity indexes are shown in Figure 3. At the beginning of storage, the thermal processing group showed a significantly lower richness value (Chao) than that observed in the control group (*p* < .05), which was primarily due to a section of microorganisms being damaged and apoptosis or a sub‐lethally injured state was caused under the high temperature conditions (Wu et al., 2020). Hernandez et al. (2019) studied the effect of heat‐shock treatment on the diversity of bacteria and observed that heat‐shock treatment immediately reduced the alpha diversity of microbial communities, which was consistent with our findings.

**Table 1:** The amount and length of 16S rRNA gene sequencing for samples during storage

Sample name	Raw sequences	Clean sequences	Sequences length (bp)
Thermal group‐0d‐1	38,588	33,914	413.42
Thermal group‐0d‐2	42,049	38,273	408.35
Thermal group‐0d‐3	36,181	34,540	409.93
Thermal group‐7d‐1	52,597	48,862	410.13
Thermal group‐7d‐2	59,081	54,501	411.18
Thermal group‐7d‐3	57,145	53,152	409.67
Thermal group‐14d‐1	33,646	29,592	409.94
Thermal group‐14d‐2	38,925	33,687	411.17
Thermal group‐14d‐3	32,224	28,425	409.26
Thermal group‐21d‐1	35,390	32,738	409.49
Thermal group‐21d‐2	50,092	45,986	410.82
Thermal group‐21d‐3	50,397	46,571	410.14
Control group‐0d‐1	40,732	39,678	408.47
Control group‐0d‐2	44,903	44,202	407.12
Control group‐0d‐3	47,815	47,020	409.23
Control group‐7d‐1	30,083	29,977	427.11
Control group‐7d‐2	32,080	31,948	427.87
Control group‐7d‐3	37,388	37,198	426.98
Control group‐14d‐1	41,975	41,731	428.47
Control group‐14d‐2	37,122	36,734	427.6
Control group‐14d‐3	56,657	56,360	428.77
Control group‐21d‐1	39,520	39,215	427.76
Control group‐21d‐2	48,710	48,292	427.85
Control group‐21d‐3	36,724	36,500	428.22

**FIGURE 4:**
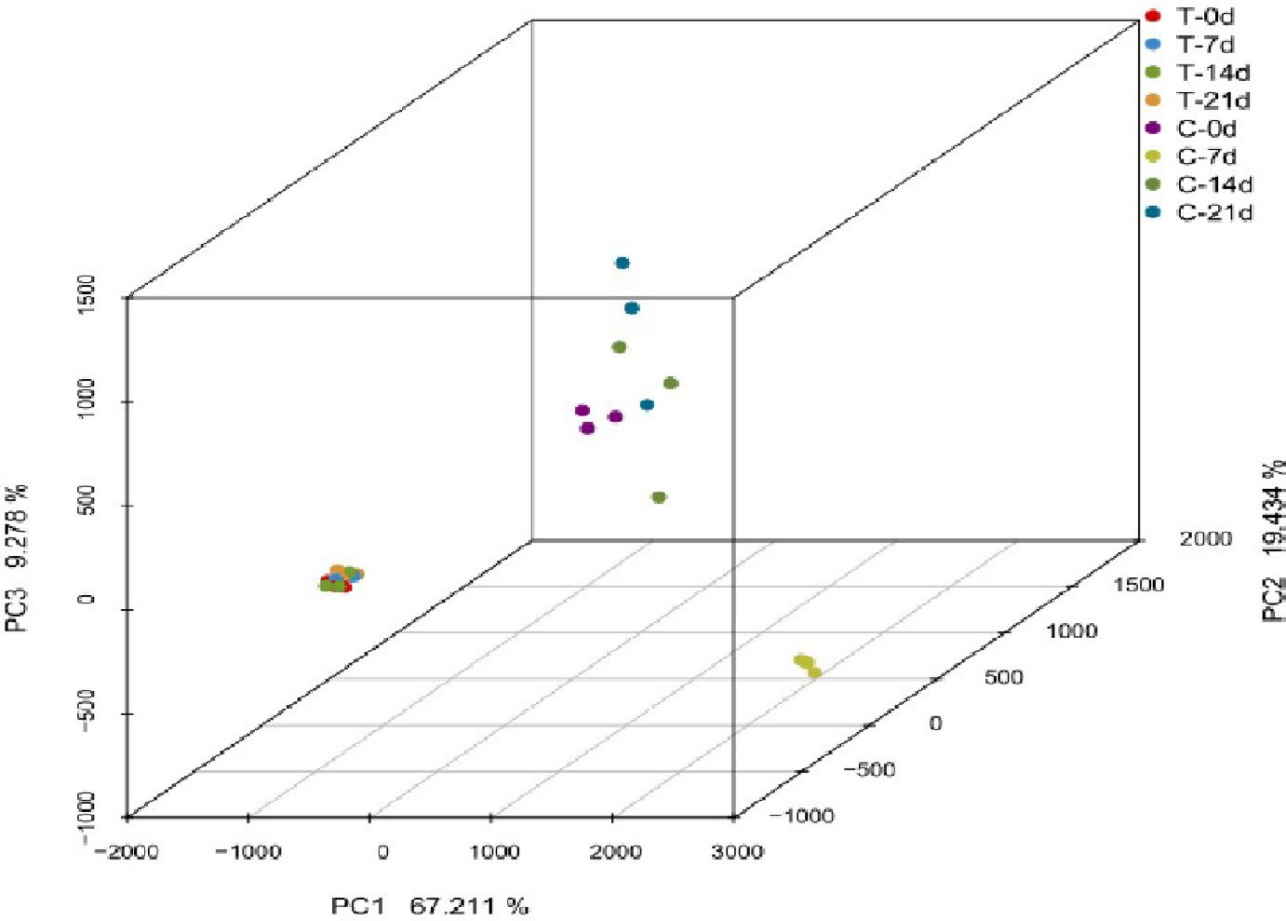
PCA of bacterial communities in the thermal treatment and control groups during storage (4°C). The numbers 0 to 21 indicate the refrigerated storage time (day). (C: Control group; T: Thermal treatment group‐121°C for 15 min)

Based on the relative abundances of OTUs sequenced from the two groups at different storage times, PCA was used to analyze the differences in bacterial community composition between the samples (Figure 4). A significant separation of bacterial communities was shown between the thermal treatment and control groups, with primary principal component (PC) scores of PC1 = 64.71%, PC2 = 16.24%, and PC3 = 8.90%. At the beginning of storage, the thermal treatment group was clearly separated from the control group, indicating that the bacterial communities changed greatly and the effect of thermal treatment on the bacterial composition was significant. For the thermal treatment group, all samples were tightly clustered together, suggesting that the difference in bacterial composition at different storage times was not significant. However, the bacterial communities of the control group at different storage days showed significant separation on the vertical axis, indicating that the effect of storage time on the bacterial community composition was significant in the control group. Riah‐Anglet et al. (2015) confirmed that heat stress induces the death of sensitive species, which in turn could promote the proliferation of surviving species, reduce competition, and facilitate their access to resources. These findings could be explained by during 4°C storage, a more stable bacterial community developed in the thermal treatment group compared with that observed in the control group.

### 3.5 Composition of bacterial communities

**FIGURE 5:**
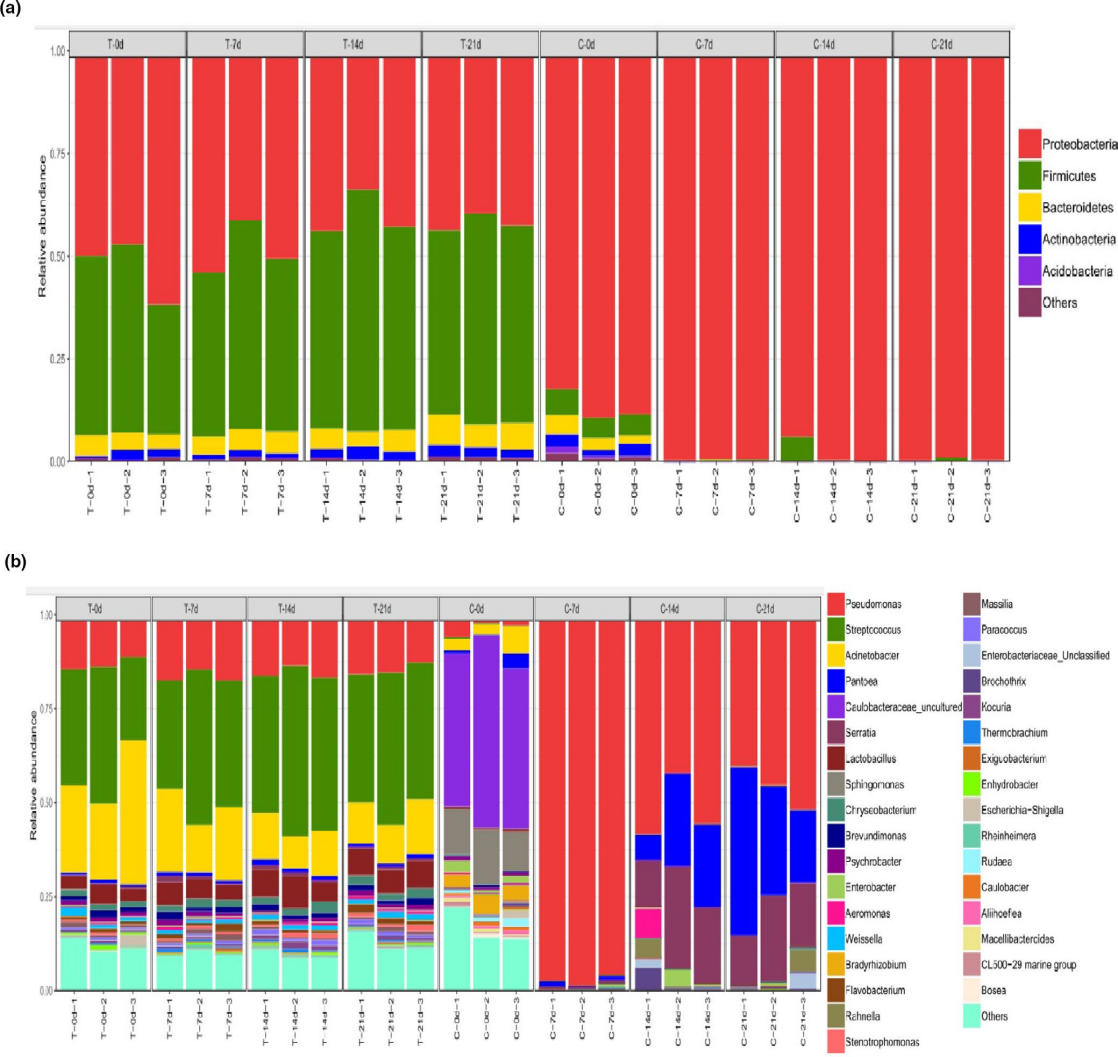
Relative abundance (%) dynamics of bacterial communities based on 16S rRNA gene sequencing results at the phylum (a) and genus (b) levels in the thermal treatment and control groups during 4°C storage. (C: Control group; T: Thermal treatment group‐121°C for 15 min)

Twenty‐one phyla were identified in the sequencing analysis, including *Proteobacteria*, *Firmicutes*, *Bacteroidetes*, *Actinobacteria*, and *Acidobacteria* (Figure 5a). In the literature, Abendroth et al. (2018) reported a heat‐resistant bacterial profile that consisted primarily of *Firmicutes*, *Bacteroidetes*, and *Proteobacteria*. *Proteobacteria* was the most predominant phyla in the thermal treatment and control groups, contributing 40.18%∼52.96% and 86.78%∼99.57% of the total OTUs, respectively. The relative abundance of *Proteobacteria* was higher in the control group than that observed in the thermal treatment group, whereas the relative abundances of *Firmicutes*, *Bacteroidetes*, and *Actinobacteria* were higher in the thermal treatment group, indicating that *Proteobacteria* could not resist high temperature compared with *Firmicutes*, *Bacteroidetes*, and *Actinobacteria*. This result may be attributed to the outgrowth of the genera *Streptococcus*, *Lactococcus*, *Chryseobacterium*, and *Flavobacterium*, which belong to the phyla *Firmicutes* and *Bacteroidetes*. For the control group, the abundance of *Proteobacteria* increased dramatically, reaching the highest levels on day 21 and contributing 99.57% of the total OTUs. *Firmicutes*, *Bacteroidetes*, *Actinobacteria*, and *Acidobacteria* displayed an opposite profile to that of *Proteobacteria*, being present at relatively high levels at the beginning of the storage period and contributing 5.39%, 3.23%, 2.51%, and 0.79% to the total OTUs, respectively, indicating that the phyla *Proteobacteria* had a greater capacity to compete for nutrients in meatballs compared with other phyla.

At the genus level, 317 different bacterial genera were detected in the samples. Figure 5b showed that the initial bacterial community in the thermal treatment group was dominated by *Streptococcus* spp. (29.80%), *Acinetobacter* spp. (27.37%), and *Pseudomonas* spp. (13.16%), whereas *uncultured‐Caulobacteraceae* spp. (44.86%), *Sphingomonas* spp. (12.33%), *Bradyrhizobium* spp. (4.26%), and *Acinetobacter* spp. (4.42%) were predominant in the control group. These findings indicate that the initial bacterial communities in meatballs were significantly altered via the thermal treatment. The reason for this result may be that the cell structures of microorganisms were destroyed in the process of the thermal treatment, resulting in a sublethal state or apoptosis of bacterial cells in meatballs. Another reason for this result may be that the enzymes participating in the growth and proliferation of microorganisms in meatballs were inactivated during the thermal treatment (Figure 4).

The relative abundance of *Pseudomonas* spp. was higher in the thermal treatment group than that observed in the control group on day 0. This result was consistent with the TVB‐N determination results, where the thermal treatment group showed a significantly higher initial TVB‐N value. The results of previous studies have indicated that the growth of *Pseudomonas* spp. during storage was associated with changes in the TVB‐N in meat products (Balamatsia et al., 2007). In addition, *Pseudomonas* spp. have been shown to possess strong proteinase and amino acid metabolic abilities in meat products (Li et al., 2019; Wang, Zhang, et al., 2017). For the control group, despite a low relative abundance of *Pseudomonas* spp. observed in the initial samples, these levels rapidly increased during storage, reaching the highest value in the meatballs on day 7 (contributing to 90.92% of the total OTUs). Subsequently, the relative abundance of *Pseudomonas* spp. continually decreased, while that of *Pantoea* spp. and *Serratia* spp. continually increased after 7 days. This transition in the bacterial community favoring facultative anaerobes could be attributed to the oxygen‐limiting conditions encountered in meat following the rapid proliferation and predominance of *Pseudomonas* spp. (Enfors & Molin, 1984). *Pseudomonas* spp. are strictly aerobic; however, oxygen in the plastic packaging bag is gradually reduced during storage. This condition was beneficial for the growth and proliferation of facultative anaerobic bacteria, such as *Pantoea* spp. and *Serratia* spp., which often contribute to meat spoilage (Gram et al., 2002; Tian et al., 2017). At the end of storage, the bacterial community was dominated by *Streptococcus* spp. (35.49%), *Acinetobacter* spp. (11.60%), and *Pseudomonas* spp. (14.27%) in the thermal treated meatballs, whereas *Pseudomonas* spp. (43.76%), *Pantoea* spp. (27.18%), and *Serratia* spp. (16.94%) dominated the control group, indicating that the thermal treatment significantly changed the bacterial communities and restrained the proliferation of potential spoilage bacteria in meatballs during storage. Accordingly, heat stress may lead to changes in microbial community composition and diversity, creating a new microbial community structure that is particularly well adapted to stress and has significant functional stability (Girvan et al., 2005; Schimel et al., 2007).

### 3.6 Functional properties of the bacterial communities

**FIGURE 6:**
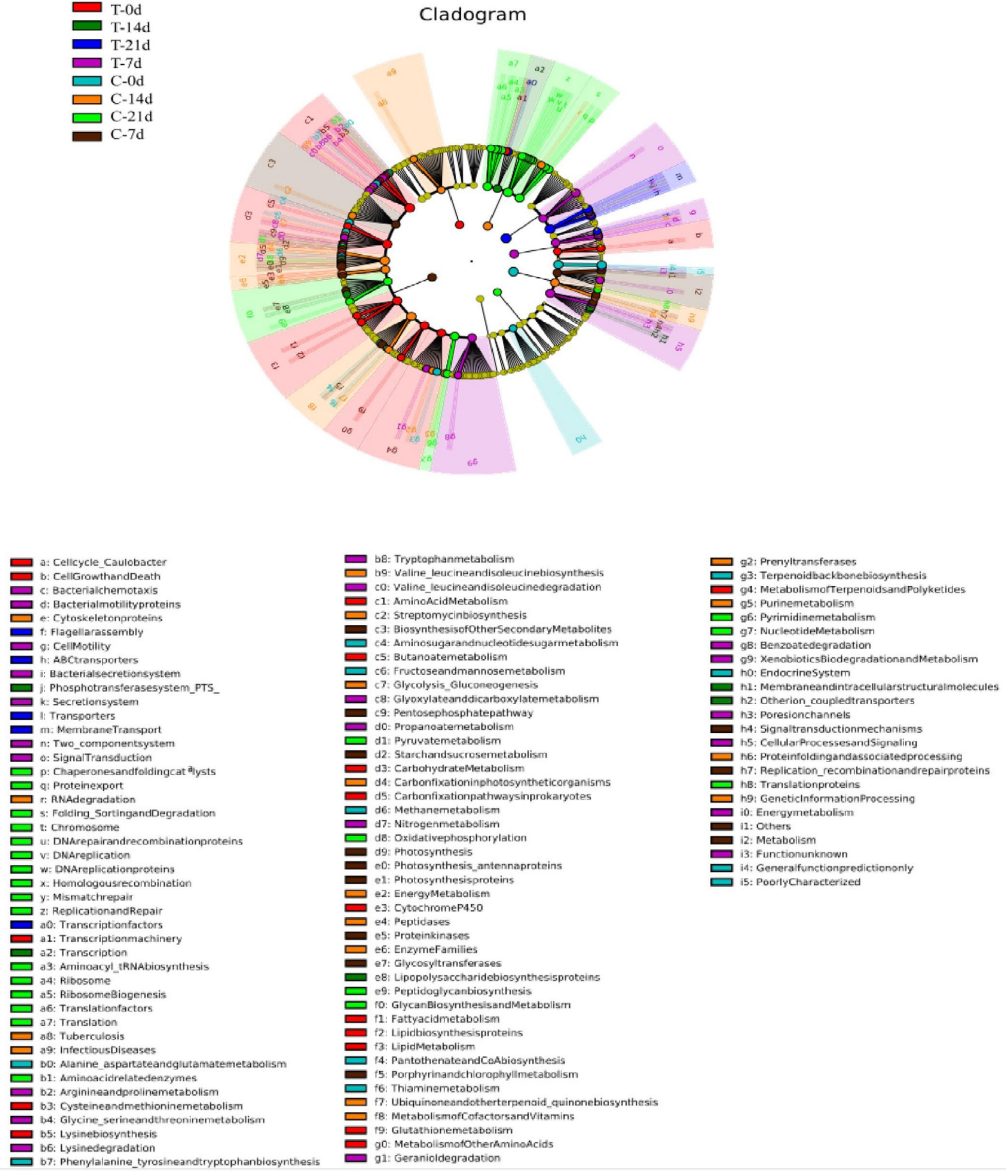
Differential phylogenetic distribution of the bacterial metabolic activities in the thermal treatment and control groups during 4°C storage. (C: Control group; T: Thermal treatment group‐121°C for 15 min)

To further investigate the changes in bacterial metabolism resulting from the thermal treatment, the relative abundances of various bacterial metabolic pathways were predicted and compared. As shown in Figure 6, metabolic pathways were abundant in the samples, suggesting that bacterial metabolism in meatballs was vigorous. Among these active metabolic pathways, carbohydrate and amino acid metabolism were the primary metabolic pathways detected in all samples, which was consistent with the results of previous studies (Li et al., 2019; Riah‐Anglet et al., 2015). More so, the biosynthesis of other secondary metabolites, carbohydrate, amino acid, lipid, and energy metabolism that were associated with bacterial activity and meat spoilage showed significant active in the control group on day 0 or day 7. This result may be due to the microorganisms participating in carbohydrate and amino acid metabolism being killed or inhibited by the thermal treatment, or possibly because the enzymes involved in the metabolic pathways mentioned above were inactivated during the thermal treatment (Figure 6).

The abundances of metabolic pathways associated with biogenic amines and sulfide formation were significantly higher in the thermal treatment group on day 0, such as those involved in phenylalanine, tyrosine and tryptophan biosynthesis metabolism, which were associated with *Enterobacteriaceae* and *Pseudomonas* spp. (Curiel et al., 2011). This result was consistent with the results of TVB‐N and the observed bacterial communities in meatballs. Furthermore, pathways involved in glyoxylate and dicarboxylate metabolism, nitrogen metabolism involving arginine, and proline metabolism related to the formation of biogenic and volatile amines were significantly abundant in the control group on day 7, a finding that was in accordance with the TVB‐N results.

## 4 CONCLUSION

The findings of this study demonstrated that thermal treatment (121°C, 15 min) could significantly decrease bacterial community diversity and the growth of potential spoilage bacteria in meatballs. In particular, thermal treatment could largely decrease the relative abundance of bacterial metabolic pathways in meatballs, such as carbohydrate, amino acid, and lipid metabolism to maintain the freshness of meatballs during storage at 4°C. This finding could contribute to a deep understanding of the influence of thermal treatment on the meat quality. Moreover, these results could provide a theoretical foundation for the development of alternative and novel nonthermal processing technologies for use in the meat industry.

## CONFLICT OF INTEREST

The authors declare that they do not have any conflict of interest.

## ETHICAL APPROVAL

This study does not involve any human or animal testing.
